# ﻿A review of the genus *Zygota* (Hymenoptera, Diapriidae) in Germany with taxonomic notes on this genus and its distinction from *Pantoclis*

**DOI:** 10.3897/zookeys.1207.121725

**Published:** 2024-07-24

**Authors:** Jeremy Hübner, Vasilisa Chemyreva, Jan Macek, Victor Kolyada

**Affiliations:** 1 Bavarian State Collection of Zoology, Munich, Münchhausenstr. 21, 81247 Munich, Germany Bavarian State Collection of Zoology Munich Germany; 2 Zoological Institute, Russian Academy of Sciences, 1 Universitetskaya Emb., St Petersburg 199034, Russia Zoological Institute, Russian Academy of Sciences St. Petersburg Russia; 3 National Museum, Department of Entomology, Praha, Czech Republic National Museum Prague Czech Republic; 4 Palaeontological Institute, Russian Academy of Sciences, Moscow 117997, Russia Palaeontological Institute, Russian Academy of Sciences Moscow Russia

**Keywords:** Checklist, DNA-barcoding, integrative taxonomy, new records, new species, new synonymy, parasitoid wasps

## Abstract

This study provides a comprehensive overview of the genus *Zygota* Förster combining DNA barcoding and current morphology. Nineteen species of *Zygota* were found throughout Germany, including the newly described species *Zygotawalli***sp. nov.** First species records for Germany are: *Zygotabalteata* Macek, 1997; *Z.comitans* Macek, 1997; *Z.spinosipes* (Kieffer, 1908); *Z.sordida* Macek, 1997; *Z.angularis* Macek, 1997 and *Z.vigil* Nixon, 1957. We also clarify diagnoses for the two related genera, *Pantoclis* Förster and *Zygota* to designate the boundaries of the *Zygota* genus and propose new synonymies: *Zygotacaligula* Buhl, 1997 is a junior synonym of *Z.congener* (Zetterstedt, 1840); *Z.reticulata* Kozlov, 1978 is a junior synonym of *Z.ruficornis* (Curtis, 1831). Thirteen species of *Zygota* sensu Nixon (1957) are transferred to the genus *Pantoclis* with the following new combinations proposed: *Zygotabrevinervis* (Kieffer, 1908) (= *Pantoclisbrevinervis* (Kieffer, 1909), **comb. nov.**); *Z.brevipennis* (Kieffer, 1908) (= *P.brevipennis* (Kieffer, 1908), **comb. nov**.); *Z.caecutiens* (Kieffer, 1908) (= *P.caecutiens* (Kieffer, 1908), **comb. nov.**); *Z.cursor* (Kieffer, 1908) (= *P.cursor* (Kieffer, 1908), **comb. nov.**); *Z.fossulata* (Thomson, 1858) (=*P.fossulata* (Thomson, 1858), **comb. nov.**); *Z.fuscata* (Thomson, 1858) (= *P.fuscata* (Thomson, 1858), **comb. no**v.); *Z.hemiptera* (Thomson, 1858) (= *P.hemiptera* (Thomson, 1858), **comb. nov.**); *Z.microtoma* (Kieffer, 1909) (= *P.microtoma* (Kieffer, 1909), **comb. nov**.); *Z.soluta* (Kieffer, 1907) (= *P.soluta* (Kieffer, 1907), **comb. nov**.); *Z.striata* (Kieffer, 1909) (= *P.striata* (Kieffer, 1909), **comb. nov**.); *Z.subaptera* (Thomson, 1858) (= *P.subaptera* (Thomson, 1858), **comb. nov**.); *Z.sulciventris* (Kieffer, 1909) (= *P.sulciventris* (Kieffer, 1909), **comb. nov.**), and *Z.unicolor* (Kieffer, 1908) (= *P.unicolor* (Kieffer, 1908), **comb. nov.**).

## ﻿Introduction

This article deals with the parasitoid wasps of the genus *Zygota* Förster (Diapriidae, Belytinae, Belytini), comprising mostly medium-sized (2.5–4.0 mm long) melanic and pubescent specimens with brightly colored appendages. The genus has 75 described species worldwide, of which most are described from the Palearctic and Nearctic (Johnson 1992; Buhl 1995, 1997, 1998; Macek 1997). Although common, little is known about their biology and their hosts. In the past, morphology-based taxonomy of *Zygota* led to confusion and many reinterpretations of the genus. The generic diagnosis, key to the species of Central Europe, and diagnostic remarks based on available types were given by Macek (1997). According to the original description of the genus given by Förster (1856)*Zygota* can be easily distinguished from other Belytinae genera by the strengthened marginalis, open radial cell, and emarginated fore tibiae in males (Förster 1856). Förster’s vague diagnosis was misinterpreted by the later authors Ashmead (1893, 1902) and Kieffer (1909), which Macek (1997, 2007) has pointed out in his revisionary works. He clarified the identity based on the designation of the neotype of *Zygotaabdominalis* (Nees, 1834), and completed a revision of available types. However, the boundary between *Zygota* and its sister genus *Pantoclis* Förster is still unclear, as some species remained falsely placed inside *Zygota*. Nixon (1957) and later Kozlov (1978) placed all Belytini species with an open radial cell and unpunctured scutellum [except some few *Belyta* species (Macek 1995)] in the genus *Zygota*. The same genus concept was applied in Johnson’s (1992) world catalog. Although the diagnosis of the genus *Zygota* was given by Macek (1997), the generic affiliation of many species was not discussed. For example, the taxonomy of the 14 species from 39 Palearctic species of *Zygota* listed by Johnson (1992) is still questionable. The genus *Pantoclis* has never been defined conclusively to exclude it from other Belytinae, because the diversity and lack of knowledge of *Pantoclis* species makes it extremely difficult to define. To understand the genus concept of *Zygota*, it must be distinguished from *Pantoclis*. We will, therefore, present a diagnosis for each.

Currently, there are 38 known species of *Zygota* in the Palearctic Region (Johnson 1992, Buhl 1995, 1997, Macek 1997). Full taxonomic treatments of the genera are given by Macek (1997) (only *Zygota*) and cataloged by Johnson (1992) (both, *Zygota* and *Pantoclis*). Macek (1997) has given a taxonomic interpretation only for 18 of these species. The present study thus aims to clarify the diagnosis of *Zygota* and the taxonomic position of the remaining 20 species, which are not discussed in Macek (1997). This revision is mostly based on material collected in Bavaria, Germany, in the framework of the German Barcode of Life (GBOL) III: Dark Taxa project (Hausmann et al. 2020). The most recent diversity evaluation that has been conducted for Germany was done over twenty years ago by Blank (2001). In his work, twenty *Zygota* taxa were recovered, of which two, *Z.excisipes* (Kieffer, 1916) and *Z.norvegica* (Kieffer, 1912), have been synonymized with *Z.excisor* (Zetterstedt, 1840) and *Z.ruficornis* (Curtis, 1831), respectively. For *Zygotasubclausa* (Kieffer, 1907), Macek (1995, 1997) proposed the new combination *Belytasubclausa* (Kieffer, 1907). In total, 19 species of *Zygota* were reliably identified for the German fauna.

## ﻿Material and methods

Most of the examined material was collected within the GBOL III project as well as from earlier collecting events in Bavaria and Baden-Wuerttemberg (Germany) led by the Bavarian State Collection of Zoology in Munich (SNSB-ZSM). Further material originates from the collection of the National Museum in Prague (NMPC) and the Russian collections in St. Petersburg (ZISP). In addition, type material from the Zoological Museum in Copenhagen (ZMUC) and the Natural History Museum (NHM) in London was examined. All specimens were morphologically identified as far as possible, including the closely related genus *Pantoclis*. Afterwards, individuals were Sanger sequenced under the usage of a voucher recovery approach. The genetic information was obtained at the Canadian Centre for DNA Barcoding (CCDB) in Guelph by the application of a voucher recovery protocol (https://ccdb.ca/). All mitochondrial CO1 sequences were aligned in MEGA11 (Tamura et al. 2021), and the alignment was then used to construct maximum likelihood trees with the online program IQ TREE version 2.0 (Trifinopoulos et al. 2016) using the default settings (1000 bootstrap alignments, substitution model: TIM+F+I+G4, 1000 iterations). Editing was done using FIGTREE version 1.4.4 (Rambaut 2010) and INKSCAPE version 1.1.1 (2021, available from: https://inkscape.org/de). Clustering and BIN-distance-analyses were conducted to infer species barriers among the CO1 barcodes using MEGA11 as well as ASAP (Puillandre et al. 2021). Suppl. material 3 gives an overview of the genetically examined material and the clustering results. All molecular data and collection metadata are publicly available on the Barcode of Life Data System (BOLD) platform (http://www.barcodinglife.org, Ratnasingham and Hebert 2007) in the dataset [DS-ZYGPAN dx.doi.org/10.5883/DS-ZYGPAN]. It is important to note that analysis was conducted on data that was downloaded from BOLD on 27 February 2024. Therefore, the results are based on the BIN-statuses of that time.

The morphological terminology and abbreviations follow those proposed by Yoder (2004) and as used in Hymenoptera Anatomy Ontology (Yoder et al. 2010); the measurements follow Yoder (2004) and Chemyreva (2015, 2018). Terms of relative position follow Goulet and Huber (1993). The terms of sculpture description follow Eady (1968). The accurate taxonomic treatments of the genera and species *Zygota* and *Pantoclis* are given in Macek (1997) and Johnson (1992). Taxa that have received an updated taxonomic treatment, such as new species or synonyms, are newly diagnosed here. Sufficiently detailed diagnoses for all other species were given by Maсek (1997). The general distribution of species was obtained and updated from Blank (2001), Wall (1963), Buhl (1995, 1997), Macek (1997), and Chemyreva et al. (2023). New records are marked with an asterisk (*). The following abbreviations for locations in Germany are used: BW= Baden-Württemberg, BY= Bavaria. Museum acronyms: SNSB-ZSM – Bavarian State Collection of Zoology, Munich; ZISP – Zoological Institute of the Russian Academy of Sciences, St. Petersburg, Russia; ZMUC – Zoological Museum, University of Copenhagen. A series of images were taken using an Olympus OM-D camera mounted on a Leica M125 C binocular and stacked using HELICON FOCUS (Version 8).

## ﻿Taxonomy

### ﻿Genus Pantoclis

Taxon classificationAnimaliaHymenopteraDiapriidae

Förster, 1856

DECB9414-E217-5F04-B765-D09DDBE1DFFC

#### Type species.

**Figure 1.**
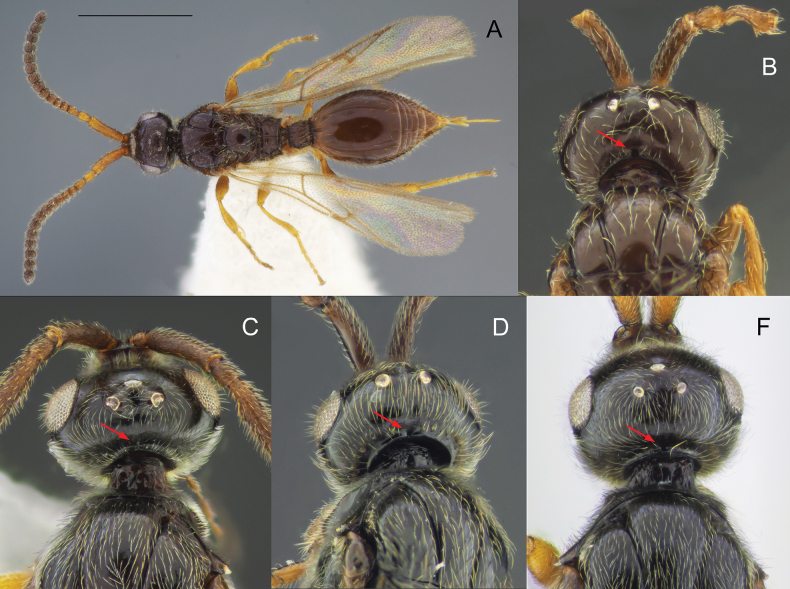
Morphological characters to identify the closely related genera *Zygota* and *Pantoclis***A, E** female **B, C, D** males **A, B***P.barycera***C***Z.walli* sp. nov. **D, E***Z.abdominalis*. Scale bars: 1 mm (**A**); 0.5 mm (**B–F**).

*Pantoclisbarycera* Förster, 1861 (Figs 1A, B, 5E).

#### Diagnosis.

**Figure 2.**
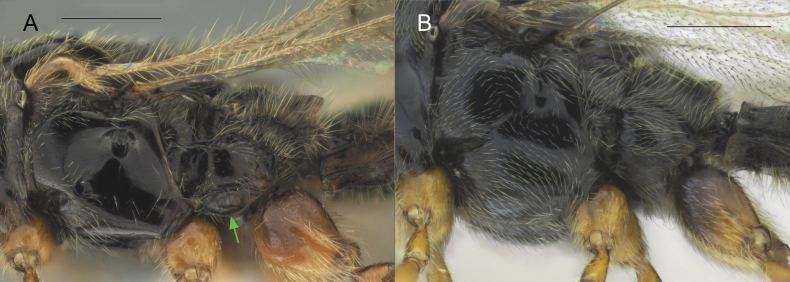
Morphological characters to identify the closely related genera *Pantoclis* (**A**) and *Zygota* (**B**) **A***Pantoclis* spp., male **B***Z.breviuscula*, male. Green arrow – submetapleural carina. Scale bars: 0.3 mm.

**Figure 3.**
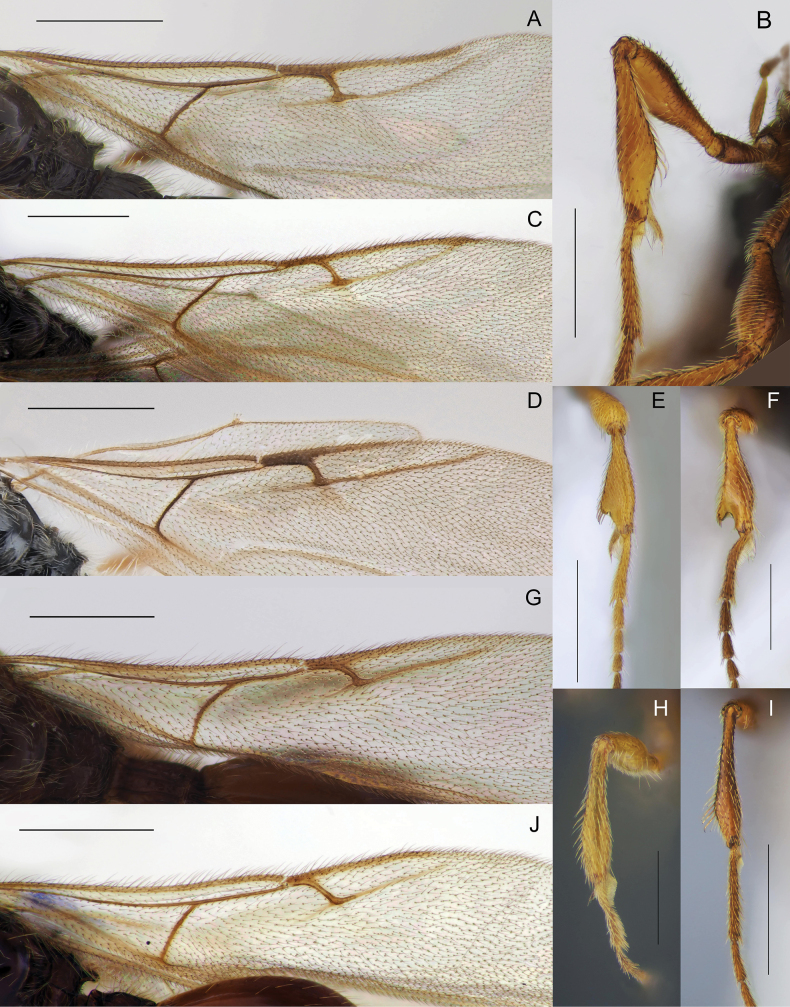
Venation (**A, C, D, G, J**) and fore tibia (**B, E, F, H, I**) morphology of males **A***Zygotabensoni***B***Z.sordida***C***Z.croton***D***Z.walli* sp. nov. **E***Z.breviuscula***F***Z.walli* sp. nov. **H***Pantoclis* sp. **I***Z.croton***G, J***Pantoclis* spp. Scale bars: 0.5 mm (**A–E**, **G, I, J**); 0.3 mm (**F, H**).

**Figure 4.**
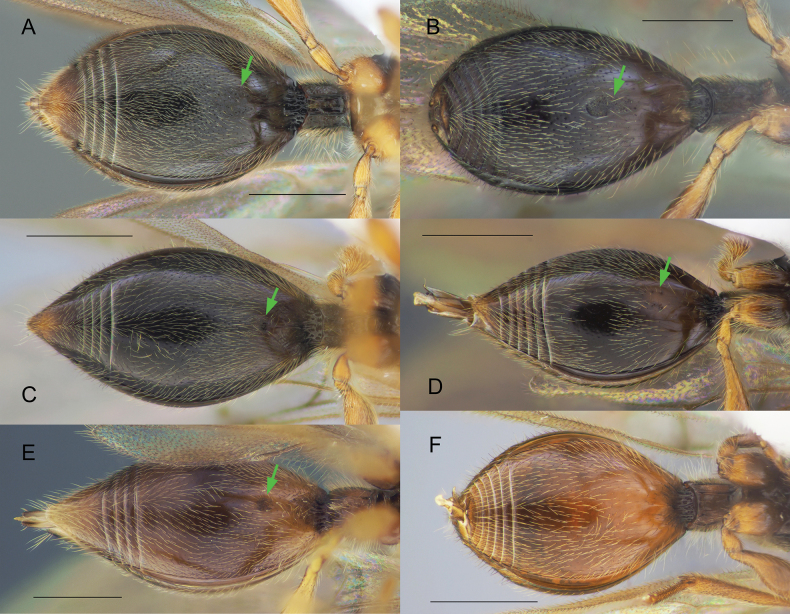
Ventral side of metasoma of females (**A, C, E**) and males (**B, D, F**) **A***Zygotabreviuscula***B***Z.abdominalis***C, D***Z.pubescence***E***Z.walli* sp. nov. **F***Pantoclis* sp. Scale bar: 0.5 mm.

**Figure 5.**
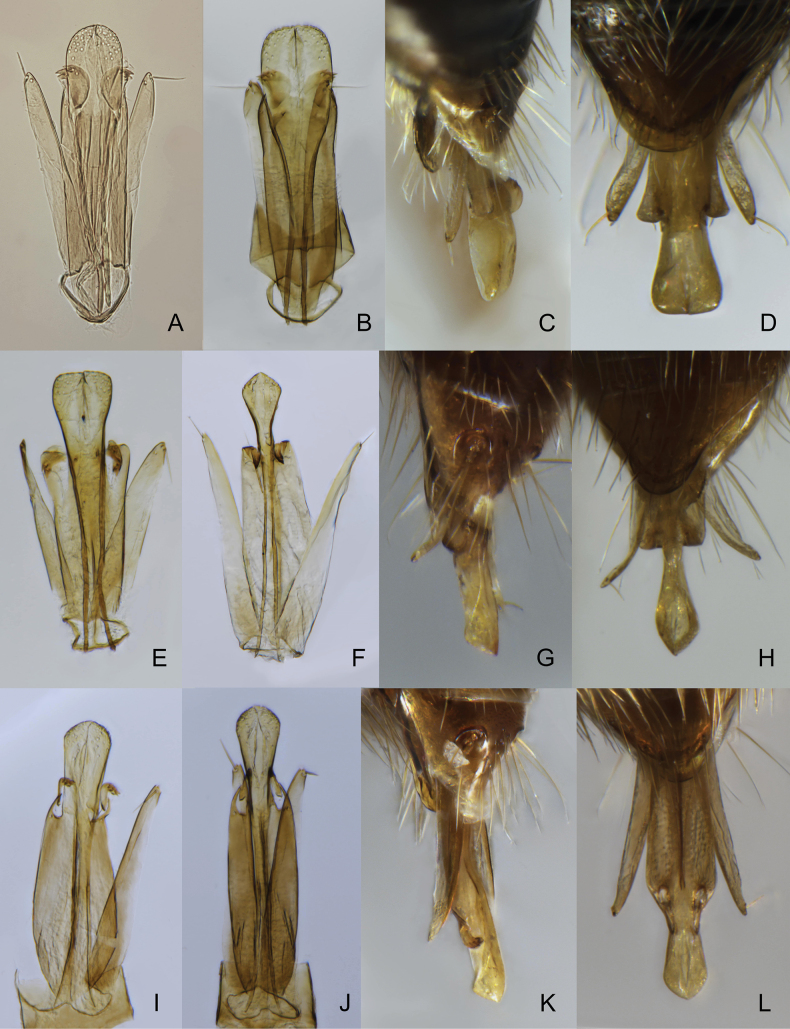
Male genitalia of *Zygota* and *Pantoclis***A***Z.walli* sp. nov. **B–D***Z.abdominalis***E***P.barycera***F–H***Pantoclis* sp. 1 **I–L***Pantoclis* sp. 2 **C, G, I, K** lateral view **A, B, D, E, F, H, J, L** ventral view.

Body black to yellowish brown; males macropterous, females alate to brachypterous or wingless; occipital carina always with occipital pit (Fig. 1B, red arrow); fore tibiae of males always unmodified with homogeneous pubescence (Fig. 3H); submetapleural carina usually present, complete (Fig. 2A, green arrow) [if submetapleural carina missing, then venation as described below]; radial cell open to closed, variable in shape (Fig. 14); radialis not parallel to parastigma [if parallel (Fig. 3G, J) then angle between stigmal and marginal veins as described below]; angle between stigmal and marginal veins 130 degrees (Fig. 3G, J) or more; S2 always smooth, without punctured area on it in anterior half (Fig. 4F); male genitalia usually slender, apex of aedeagus distinctly convex (Fig. 5I–L), lanceolate (Fig. 5F–L), rather truncate (Fig. 5E) [if genitalia short and stout with rounded aedeagus then fore wing with a closed radial cell], digitus usually diminished (Fig. 5E–L) [if not then fore wing with closed radial cell]; ovipositor usually long, at least as long as length of T2 [if ovipositor short then fore wing with closed radial cell].

### ﻿Genus Zygota

Taxon classificationAnimaliaHymenopteraDiapriidae

Förster, 1856

80850927-2072-5389-B22A-DD3C9DA00D71

Zygota Förster, 1856: 128, 131, 133, 135. Type species: Belytaabdominalis Nees van Esenbeck, designated by Ashmead (1893).
Carinia Kieffer, 1905: 140. Type: Carinianitida Kieffer, by monotypy and original designation. Synonymized with Aclista Förster by Kieffer (1910), with Zygota Förster by Muesebeck (1951).

#### Diagnosis.

Body always black (only metasoma very rarely brown); males and females alate; occipital carina with or without occipital pit (Fig. 1C–F, red arrows); fore tibiae modified in some males or bear several stiff setae (Fig. 3B, E, F, I); submetapleural carina missing (Fig. 2B), or reduced; radial cell long, open at apex (except *Z.croton* Fig. 3C); radialis long and almost parallel to parastigma (Fig. 3D); angle between stigmal and marginal veins at most 120 degrees; some species with small depression (Fig. 4B) or micro-puncture sculpture on S2 in anterior half (Fig. 4A, C–E, green arrows); male genitalia short and stout, apex of aedeagus truncate or rounded, digitus large (Fig. 5A–D); complete ovipositor always short, at most as long as pygidium (8^th^ + 9^th^ tergite above, 7^th^ sternite below).

#### Remarks.

Based on the diagnoses and original descriptions of the species *Zygotacaecutiens* (Kieffer, 1908), *Z.hemiptera* (Thomson, 1858), *Z.microtoma* (Kieffer, 1909), *Z.soluta* (Kieffer, 1907) and the generic diagnoses of *Zygota* and *Pantoclis*, these four species should be excluded from *Zygota* and considered as part of *Pantoclis*; *Pantocliscaecutiens* (Kieffer, 1908), comb. nov., *P.hemiptera* (Thomson, 1858), comb. nov., *P.microtoma* (Kieffer, 1909), comb. nov. and *P.soluta* (Kieffer, 1907), comb. nov. Moreover, based on the study of the type specimens the following species are transferred from *Zygota* to *Pantoclis*: *Pantoclisbrevinervis* (Kieffer, 1909), comb. nov., *P.brevipennis* (Kieffer, 1908), comb. nov., *P.cursor* (Kieffer, 1908), comb. nov., *P.fossulata* (Thomson, 1858), comb. nov., *P.fuscata* (Thomson, 1858), comb. nov., *P.striata* (Kieffer, 1909), comb. nov., *P.subaptera* (Thomson, 1858), comb. nov., *P.sulciventris* (Kieffer, 1909), comb. nov. and *P.unicolor* (Kieffer, 1908), comb. nov. (see also Suppl. material 2 for an overview of type locations and the museums where the specimens are stored).

### ﻿ Zygota abdominalis

Taxon classificationAnimaliaHymenopteraDiapriidae

(Nees, 1834)

E3A2B005-9F02-5ECE-9E90-630E89F0CE5A

Figs 1D, E , 4B , 5B–D

Belyta abdominalis Nees, 1834: 344, male.
Zygota abdominalis : Macek 1997: 37, male, female, neotype designation.

#### BOLD BIN.

BOLD:AEJ6743.

#### Material examined.

Germany: BY: NGS Schwarzes Moor, 09-Aug-2017, 1 ♂; Paehl, 21-Mar-2020, 24-Apr-2020, 4 ♂; Ammer mountains, 27-Aug-2016, 1 ♂; Kehlheim, 10-Apr-2017, 1 ♂; Balderschwang, 21-Sept–12-Oct-2017, 1 ♀, 4 ♂; Kehlheim, 23-Aug–08-Sept-2017, 1 ♂; NSG Romberg, 18-May–09-Jun-2018, 2 ♂; Paehl, 24-Apr–08-May-2020, 7 ♂; Rhoen mountains, 27-Jun–11-Jul-2018, 2 ♂; Ketterschwang, 01–16-Jul-2019, 1 ♂; Grafenreuth, 01–15-Jul-19, 4 ♂. BW: Malsch, 27-Jun–09-Jul-2011, 2 ♂; Gaggenau-Sulzbach, 02–21-Aug-2011, 1 ♀.

#### Distribution.

Europe: Czech Republic, Germany, Poland, Russia (European part).

### ﻿ Zygota angularis

Taxon classificationAnimaliaHymenopteraDiapriidae

Macek, 1997

72337A73-EEAC-585C-B7F9-8E6A61E31F69

Zygota angularis Macek, 1997: 54, male, female.

#### BOLD BIN.

BOLD:ACQ5437.

#### Material examined.

Germany: BY: Mittenwald, 30-Jul-2021, 1 ♂; Rhoen mountains, 11-Jul-2018, 3 ♂.

#### Distribution.

Europe: Czech Republic, Germany*, Slovenia.

### ﻿ Zygota balteata

Taxon classificationAnimaliaHymenopteraDiapriidae

Macek, 1997

1535DA15-9513-54E4-A2B9-5ED93071CAB7

Zygota balteata Macek, 1997: 40, male, female.

#### BOLD BIN.

No BIN.

#### Material examined.

Germany: BY: NSG Fellinger Mountain, 08-Jun-2013, 1 ♀, Grafenaschau, 2013, 1 ♀.

#### Distribution.

Europe: Czech Republic, Germany*, Slovenia.

### ﻿ Zygota breviuscula

Taxon classificationAnimaliaHymenopteraDiapriidae

(Thomson, 1858)

957C147D-93BB-5744-ADBE-3AB1B99A2697

Figs 2B , 3E , 4A

Belyta breviuscula Thomson, 1858: 176, female.
Aclista sulcata Kieffer, 1909. Synonymized by Macek (1997).
Zygota larides Nixon, 1957. Synonymized by Macek (1997).

#### BOLD BIN.

No BIN.

#### Material examined.

Germany: BY: Ammer mountains, 05-Oct-2016, 1 ♀; Oberstdorf, 10–24-Jul-2016, 24-Jul-2016 and 28-Jun-2016, 15 ♂.

#### Distribution.

Europe: Austria, Czech Republic, Germany, Hungary, Italy, Russia (European part), Slovenia, Sweden.

### ﻿ Zygota claviscapa

Taxon classificationAnimaliaHymenopteraDiapriidae

(Thomson, 1858)

6DEFF4D9-438F-5F21-8690-27B1B4163F55

Belyta claviscapa Thomson, 1858: 175, female, male.
Aclista brevicornis Kieffer, 1909. Synonymized by Macek (1997).

#### BOLD BIN.

No BIN.

#### Material examined.

Germany: BY: Garmisch-Partenkirchen, 2–13-Aug-2018, 3 ♂; Oberstdorf, 28-Jun-2016, 2 ♂; Grafenreuth, 1–15-Jul-19, 1 ♂.

#### Distribution.

Europe: Austria, Czech Republic, England, Germany, Hungary, Ireland, Poland, Russia (European part), Scotland, Slovenia, Sweden.

### ﻿ Zygota comitans

Taxon classificationAnimaliaHymenopteraDiapriidae

Macek, 1997

A935DE49-48E9-54B2-9B30-9D68ED0EAE3F

Zygota comitans Macek, 1997: 47, female, male.

#### BOLD BINs.

BOLD:AEL3896, BOLD:AEJ0891.

#### Material examined.

Germany: BY (BOLD:AEL3896): Moos, Isarmuendung, Hartholzauwald, 16-Jun-2021, 1 ♂; Chiemgauer Alpen, Ruhpolding, Fischbach, 02-Aug-2016, 1 ♂; Paehl, 24-Apr-2020, 1 ♂. BY (BOLD:AEJ0891): Berchtesgaden, Bartholomae, NP Berchtesgarden, Wald, 13-Sep-2017, 1 ♀; Gaggenau, Michelbach, 21-Aug-2011, 1 ♀; Paehl, Niedermoor w Goasl, 19-Sep-2020, 1 ♀. BY (unsequenced material): Rhoen mountains, 27-Jun–11-Jul-2018, 3 ♂; Grafenaschau, 2013, 1 ♂; Oberstdorf, 28-Jun-2016, 1 ♂.

#### Distribution.

Finland, Germany*, Poland, Slovenia, Sweden.

### ﻿ Zygota congener

Taxon classificationAnimaliaHymenopteraDiapriidae

(Zetterstedt, 1840)

D552001D-904D-5906-883C-E967B78ABC41

Figs 6A‒F , 7A‒F

Psilus (Belyta) congener Zetterstedt, 1840: 415, female, male.
Zygota caligula Buhl, 1997: 53, female. Syn. nov.

#### BOLD BIN.

BOLD:AAI8609.

#### Material examined.

**Figure 6.**
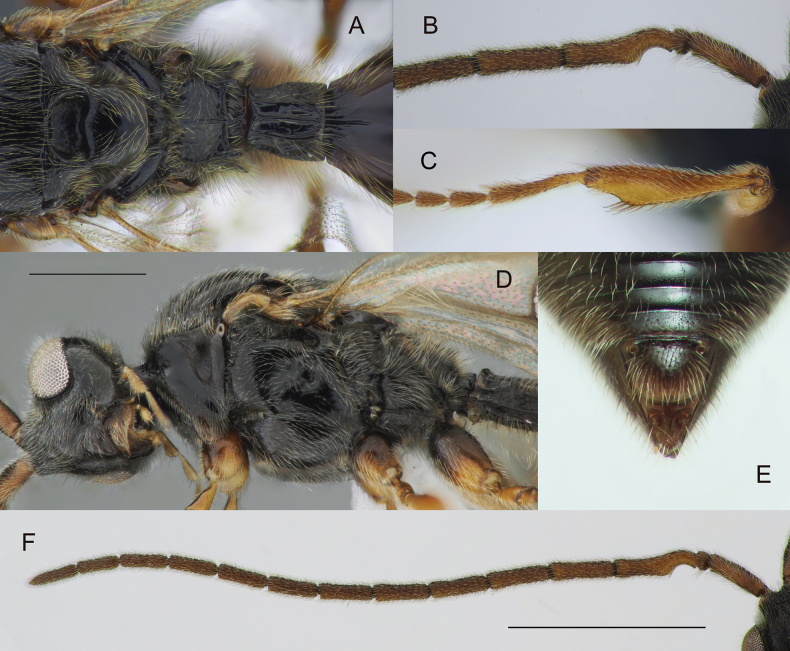
*Zygotacongener*, male (**B–D, F**) and female (**A, E**) **A** mesosoma and petiole in dorsal view **B** A1–A5 in ventral view **C** fore tibia **D** head and mesosoma in lateral view **E** apex of metasoma in dorsal view (*Z.caligula* Buhl, holotype) **F** antennae in ventral view. Scale bars: 0.5 mm (**D**); 1 mm (**F**).

***Holotype*** of *Zygotacaligula*: Norway: Mosvik, 14-Aug-1994, “MT. JT:19”, “Smafa”, P.N. Buhl det. 1996, Holotype, ZMUC 00021242, Zygotacaligula, 1 ♀. Germany: BY: Garmisch-Partenkirchen, 02-Aug-2018, 13-Aug-2018, 09-Oct-2018, 4 ♂; Grafenaschau, 2013, 1 ♂ (Fig. 6E)

#### Diagnosis.

**Figure 7.**
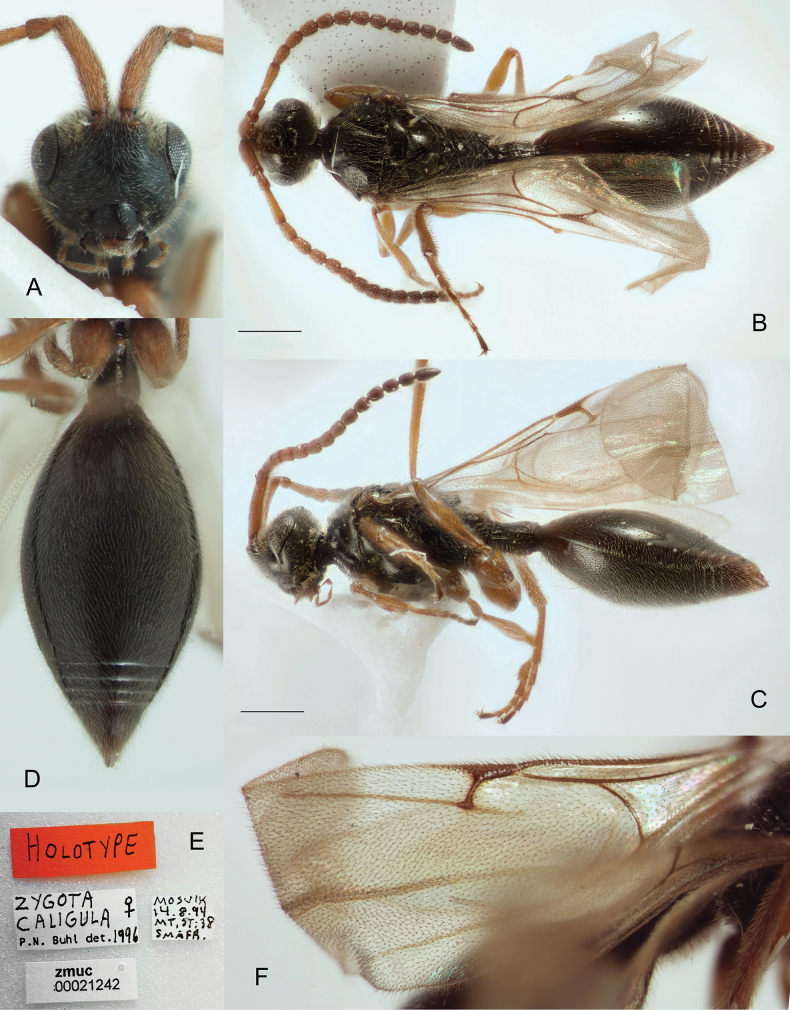
Holotype of the *Zygotacaligula* Buhl **A** face **B** body in dorsal view **C** body in lateral view **D** metasoma, ventral view **E** type material labels **F** fore wing venation. Scale bar: 0.5 mm.

**Both sexes**: postmarginal vein distinctly shorter than radial cell length (Fig. 7F); occipital pit present; mesopleuron with only small bare area medially or entirely pubescent (Fig. 6D); axillar depression with scattered setae and only 2 verriculate tubercles; propodeal spiracle distinctly enlarged (Fig. 6A); base of T2 with lateral corners (Fig. 6A); S2 without micro-puncture sculpture anteriorly. **Female**: female antenna with A6‒A14 about 1.25 times as long as wide (Fig. 7B, C); T2 punctuated (Fig. 7B, C); T8 (apical) with median keel between cerci (Fig. 6E). **Male**: A3 strongly emarginate (Fig. 6B); fore tibia slightly modified, weakly humped interiorly, entirely pubescent and with a row of enlarged setae along its inner side (Fig. 6C); genitalia as in *Z.walli* sp. nov. and *Z.abdominalis* (Fig. 5A‒D), digitus armed with 3 or 4 teeth.

#### Remarks.

The female of *Zygotacongener* is best recognized by the large propodeal spiracles (Fig. 6A) and the sharp median keel between the cerci on the apical tergite of the female (Fig. 6E). These two characters, together with other peculiarities of the morphology of *Z.congener*, correspond to the characters of the holotype of *Z.caligula* Buhl. For this reason, *Z.caligula* is considered here to be a junior synonym of *Z.congener*.

#### Distribution.

Austria, Czech Republic, Denmark, Finland, Germany, Russia (European part), Slovenia, Sweden.

### ﻿ Zygota croton

Taxon classificationAnimaliaHymenopteraDiapriidae

Nixon, 1957

27B10EA7-F82A-57FA-9114-BC29113F5AD0

Fig. 3C, I

Zygota croton Nixon, 1957: 29, 62, male, female.

#### BOLD BIN.

BOLD:AEK1965.

#### Material examined.

Germany: BY: Mittenwald, 30-Jul-2021, 1 ♂; Garmisch-Partenkirchen, 05-Jul-2018, 18-Jul-2018, 02-Aug-2018, 13-Aug-2018, 1 ♀, 16 ♂; Oberstdorf, 10-24-Jul-2016, 1 ♂.

#### Distribution.

Europe: Austria, Czech Republic, France, Germany, Russia (European part), Scotland, Slovenia, Sweden.

### ﻿ Zygota excisor

Taxon classificationAnimaliaHymenopteraDiapriidae

(Zetterstedt, 1840)

FE7786C0-A9E8-5443-BE2C-7AC94AB306B3

Psilus (Belyta) excisor Zetterstedt, 1840: 415, male.
Aclista lanceolata Kieffer, 1909. Synonymized by Macek (1997).
Aclista lanceolata var. fuscicornis Kieffer, 1909. Synonymized by Macek (1997).
Aclista semirufa Kieffer, 1909. Synonymized by Macek (1997).
Aclista (Zygota) excisipes Kieffer, 1908. Synonymized by Macek (1997).

#### BOLD BIN.

No BIN.

#### Material examined.

Germany: BY: Lohr am Main, 06-Sep-2016, 1 ♂; Rhoen mountains, 11-Jul-2018, 1 ♂; Oberstdorf, 28-Jun-2016, 1 ♀; Ruhpolding, 19-Jul-2016, 1♂; Garmisch-Partenkirchen, 13-Aug-2018, 1 ♂.

#### Distribution.

Europe: Austria, Czech Republic, Germany, Hungary, Italy, Poland, Russia (European part), Slovenia, Sweden.

### ﻿ Zygota nigra

Taxon classificationAnimaliaHymenopteraDiapriidae

(Thomson, 1859)

68287CA0-DE94-5D45-8325-9AACDED0719A

Belyta nigra Thomson, 1859: 175, female.
Aclista lanceolata Kieffer, 1909. Synonymized by Macek (1997).

#### BOLD BIN.

BOLD:AEJ4945.

#### Material examined.

Germany: BY: Mittenwald, 30-Jul-2021, 3 ♂, 1 ♂; Garmisch-Partenkirchen, 05-Jul-2018, 13-Aug-2018, 11-Sep-2018, 3 ♂.

#### Distribution.

Europe: Algeria, Czech Republic, Germany, Russia (European part), Slovenia, Sweden.

### ﻿ Zygota parallela

Taxon classificationAnimaliaHymenopteraDiapriidae

(Thomson, 1859)

6391D137-13FC-5915-9E4A-DF411BC28FF7

Belyta parallela Thomson, 1859: 175, male.
Aclista macroneura Kieffer, 1909. Synonymized by Macek (1997).

#### BOLD BINs.

BOLD:ACU1498, BOLD:AEJ0893.

#### Material examined.

(BOLD:ACU1498) Germany: BY: Berchtesgaden, 11-Jun-2017, 3 ♂; Rhoen mountains, 27-Jun–11-Jul-2018, 2 ♀, 1 ♂; NSG Metzgergraben, 25-Jun-2016, 1 ♂; NSG Metzgergraben, 10–25-Jun-2016, 10 ♀, 37 ♂; Oberstdorf, 24-Jul-2016, 1 ♀, 17 ♂; Oberstdorf, 28-Jun-2016, 12 ♂; Siegenburg, 08–26-May-2017, 4 ♂; Grafenreuth, 01–15-Jul-2019, 1 ♀, 1 ♂; Paehl, 24-Apr-08-May-2020, 6 ♂; Rhoen mountains, 27-Jun-18-Jul-2018, 10 ♂; NSG “Schwarzes Moor”, 26-Jun–18-Jul-2017, 4 ♂. Material examined (BOLD:AEJ0893). Germany: BY: Sugenheim, 24-May-2021, 1? (ZSM-HYM-42355-A04); Garmisch-Partenkirchen, 13-Aug-2018, 1 ♀; Markt Nordheim, 02-May-2019, 1 ♂.

#### Distribution.

Europe: Austria, Czech Republic, Germany, Hungary, Poland, Scotland, Slovenia, Sweden.

### ﻿ Zygota praetor

Taxon classificationAnimaliaHymenopteraDiapriidae

Nixon, 1957

623010D8-A52B-59C7-9421-217B8C21748A

Zygota praetor Nixon, 1957: 58, 62, male, female.

#### BOLD BIN.

No BIN.

#### Material examined.

Germany: BY: Oberstdorf, 24-Jul-2016, 1 ♂.

#### Distribution.

Europe: Czech Republic, Denmark, Germany, Ireland, Slovenia, Sweden.

### ﻿ Zygota pubescens

Taxon classificationAnimaliaHymenopteraDiapriidae

(Kieffer, 1909)

04EE14C8-9168-554B-B3A0-3445E679181F

Fig. 4C, D

Aclista lanceolata var. pubescens Kieffer, 1909: 473. Female.
Pantoclis cameroni : Kieffer 1907. Synonymized by Macek (1997).

#### BOLD BIN.

BOLD:ACC4346.

#### Material examined.

Germany: BY: Mittenwald, 13-Jul-2021, 1 ♂; Paehl, 21-Mar-2020, 24-Apr–08-May-2020, 2 ♀, 1 ♂; Ketterschwang, 01–16-Jul-2019, 1 ♂; Balderschwang, 21-Sep–12-Oct-2017, 3 ♂; Rhoen mountains, 27-Jun–11-Jul-2018, 5 ♂; Garmisch- Partenkirchen, 02-Aug-2018, 1 ♀; NSG Allacher Lohe, 01-Sep-2021, 1 ♂; NSG Allacher Lohe, Munich, 08-Jun–23-Jun-2021, 3 ♂; NSG Metzgergraben, 10–25-Jun-2016, 2 ♂; Siegenburg 08–26-May-2017, 2 ♂; Oberstdorf, 10–24-Jul-2016, 2 ♂.

#### Distribution.

Europe: Austria, Czech Republic, Germany, Italy, Russia (European part), Scotland, Slovenia, Sweden.

### ﻿ Zygota ruficornis

Taxon classificationAnimaliaHymenopteraDiapriidae

(Curtis, 1831)

48780E4A-2C92-5786-9ADE-5F5820181A60

Fig. 8A–I

Cinetus ruficornis Curtis, 1831: 380, female.
Aclista dentatipes Kieffer, 1908: 447. Synonymized by Macek (1997).
Aclista norvegica Kieffer, 1912: 20. Synonymized by Macek (1997).
Zygota reticulata Kozlov, 1978: 575, female. Syn. nov.

#### BOLD BINs.

BOLD:AEX2887, BOLD:AEK5610, BOLD:AEY0233.

#### Material examined.

**Figure 8.**
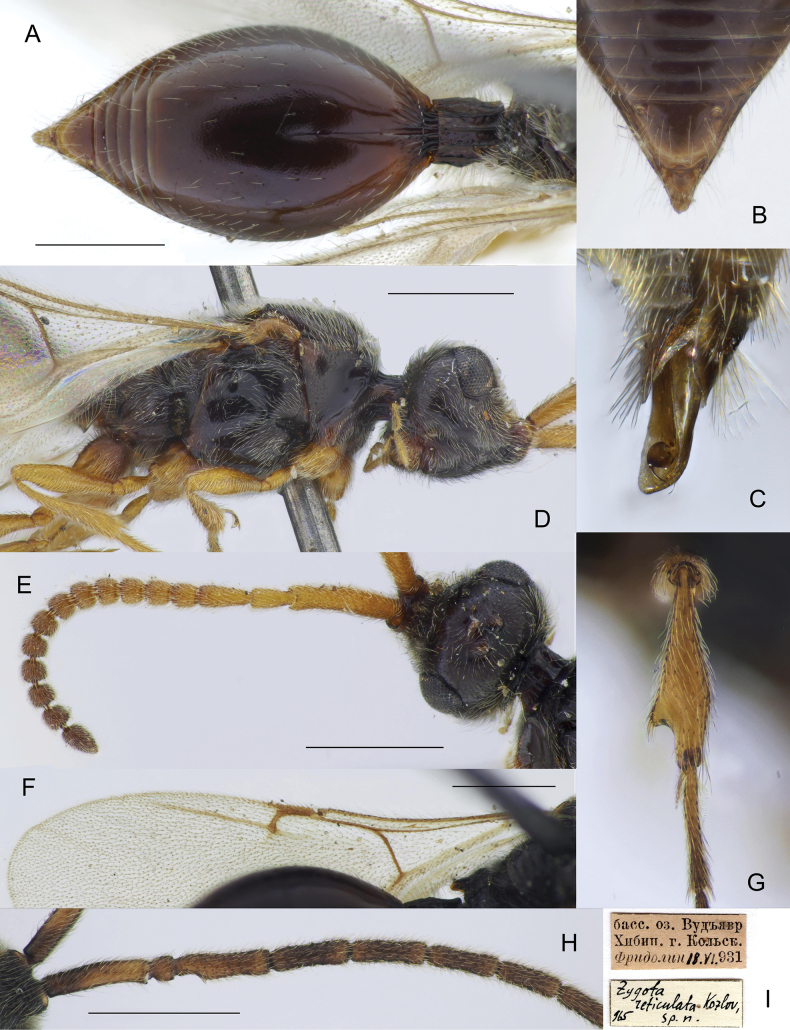
*Zygotaruficornis* male (**C, G, H**) and female (*Z.reticulata* Kozlov, holotype) (**A**, **B, D, E, F**) **A** metasoma, dorsal view **B** apex of metasoma, dorsal view **C** genitalia, lateral view **D** head and mesosoma, lateral view **E** antennae, dorsal view **F** fore wing **G** fore tibia **H** antenna, proximal part **I** label of the holotype. Scale bar: 0.5 mm.

***Holotype*** of *Zygotareticulata*: Russia: Kola Peninsula, Lake Vud’yavr basin, Khibiny Mountains, Kol’sk Mt., 18-Jun-1931, Fridolin leg., 1 ♀ (Fig. 8I). Germany: BY (BOLD:AEX2887): Mittenwald, 30-Jul-2021, 1 ♂. BY (BOLD:AEY0233): Paehl, 08-May-2020, 1 ♂; Mittenwald, 13-Jul-2021, 1 ♂. BY (BOLD:AEK5610): Mittenwald, 30-Jul-2021, 3 ♂; Garmisch-Partenkirchen, 18-Jul-2018, 02-Aug-2018, 4 ♂. BY (unsequenced material): Garmisch-Partenkirchen, 05-Jul-2018, 18-Jul-2018, 02-Aug-2018, 09-Oct-2018, 4 ♂; Garmisch-Partenkirchen, 13-Aug-2018, 1 ♀, 9 ♂; Bad Windsheim, 12-Jul-2020, 1 ♂; Aub, 21-May-2020, 1 ♂; Grettstadt, 20-May-2020, 1 ♀; Oberstdorf, 28-Jun-2016, 1 ♀, 6 ♂; Rhoen mountains, 27-Jun-11–Jul-2018, 21 ♂; Grafenreuth, 01–15-Jul-2019, 7 ♂; NSG Metzgergraben, 10–25-Jun-2016, 15 ♂; NSG Romberg, 18-May–09-Jun-2018, 3 ♂; Ketterschwang, 01–16-Jul-2019, 3 ♂; Siegenburg, 08–26-May-2017, 2 ♂; Garmisch-Partenkirchen, 02–13-Aug-2018, 2 ♂; NSG “Schwarzes Moor”, 26-Jun–18-Jul-2017, 2 ♂; Paehl, 24-Apr–08-May-2020, 2 ♂; Kehlheim, 29-Jun–13-Jul-2017, 1 ♂; Lohr a. M., 03–14-Jun-2018, 1 ♂; NSG Allacher Lohe, Munich, 08–23-Jun-2021, 1 ♂. BW (unsequenced material): Malsch, 27-Jun–09-Jul-2011, 1 ♀, 4 ♂.

#### Diagnosis.

**Both sexes**: postmarginal vein distinctly shorter than radial cell length (Fig. 8F); occipital pit present; mesopleuron with only small bare area on it medially or entirely pubescent (Fig. 8D); axillar depression with scattered setae and only 2 verriculate tubercles; base of T2 with small lateral corners (Fig. 8A). **Female**: T2 finely granulate (Fig. 8A); Т8 without transverse or elongate carinae on it (Fig. 8B); S2 with a small pit in anteriorly half (as in Fig. 4C, green arrow). **Male**: A3 weakly emarginate (Fig. 8H); fore tibia broadened, with sharp projection and a row of strong setae on the top of it, bare at the apex on its anterior surface (Fig. 8G); S2 with a small area of micropuncture in anteriorly half (as in Fig. 4E, green arrow); digitus armed with 1 long curved spine; spine extending from digitus at significant angle and not pushed towards it (Fig. 8C).

This species is very similar to *Z.pubescens* except as follows: female antenna stout, with A6‒A14 distinctly transverse (A6‒A14 subquadrate in *Z.pubescens*); male genitalia armed with a spine, which extends from digitus at significant angle (this spine pushed towards digitus in *Z.pubescence*). Both species are very common in Germany.

#### Distribution.

Europe: Austria, Czech Republic, France, Germany, Hungary, Norway, Poland, Russia (European part), Scotland, Slovenia.

### ﻿ Zygota sordida

Taxon classificationAnimaliaHymenopteraDiapriidae

Macek, 1997

5BBFFD56-57D3-5DB1-AD00-D36DC0AF7A2E

Fig. 3B

Zygota sordida Macek, 1997: 11, female, male.

#### BOLD BIN.

No BIN.

#### Material examined.

Germany: BY: Paehl, 24-Apr-2020, 1 ♂; Oberstdorf, 10–24-Jul-2016, 1 ♂.

#### Distribution.

Europe: Austria, Czech Republic, Germany*, Slovenia.

### ﻿ Zygota spinosa

Taxon classificationAnimaliaHymenopteraDiapriidae

(Kieffer, 1908)

C1D21897-C1F1-509D-969A-B52069656C2B

Aclista (Zygota) spinosa Kieffer, 1908: 448, male.
Zygota comes Nixon, 1957: 63, male. Synonymized by Macek (1997).
Zygota loris Nixon, 1957: 59, female. Synonymized by Macek (1997).

#### BOLD BINs.

BOLD:AEL5584, BOLD:AER0775.

#### Material examined.

Germany: BY (BOLD:AEL5584): Mittenwald, 13-Jul-2021, 30-Jul-2021, 2 ♂; Garmisch-Partenkirchen, 02-Aug-2018, 13-Aug-2018, 11-Sep-2018, 5 ♀, 6 ♂. BY (BOLD:AER0775): Garmisch-Partenkirchen, 02-Aug-2018, 1 ♂; Garmisch-Partenkirchen, 11-Sept-2018, 1 ♂.

#### Distribution.

Austria, Czech Republic, Germany, Slovenia, Switzerland.

### ﻿ Zygota spinosipes

Taxon classificationAnimaliaHymenopteraDiapriidae

(Kieffer, 1908)

399F7C65-0D3A-5E3D-A5E5-702BC59BFD82

Aclista (Zygota) spinosipes Kieffer, 1908: 446, male.

#### BOLD BIN.

BOLD:ACK3325, BOLD:AEY9457.

#### Material examined.

Germany: BY (BOLD:ACK3325): Mittenwald, 30-Jul-2021, 1 ♀, 1 ♂; Garmisch-Partenkirchen, 11-Sep-2018, 2 ♀; NP Berchtesgaden, 09-Aug-2017, 1 ♀. BY (BOLD:AEY9457): Garmisch-Partenkirchen, 13-Aug-2018, 1 ♀; Mittenwald, 30-Jul-2021, 1 ♂, 1 ♀. BY (unsequenced material): Oberstdorf, 28-Jun-2016, 1 ♀.

#### Distribution.

Europe: Czech Republic, Germany*, Italy, Russia (European part), Sweden.

### ﻿ Zygota vigil

Taxon classificationAnimaliaHymenopteraDiapriidae

Nixon, 1957

D7BC11A8-DFF3-502F-A6A7-74E698786ADD

Figs 9A–C , 10A–G

Zygota vigil Nixon, 1957: 65, male.

#### BOLD BIN.

No BIN.

#### Material examined.

Germany: BY: Garmisch-Partenkirchen, 18-Jul-2018, 1 ♂.

#### Diagnosis.

**Figure 9.**
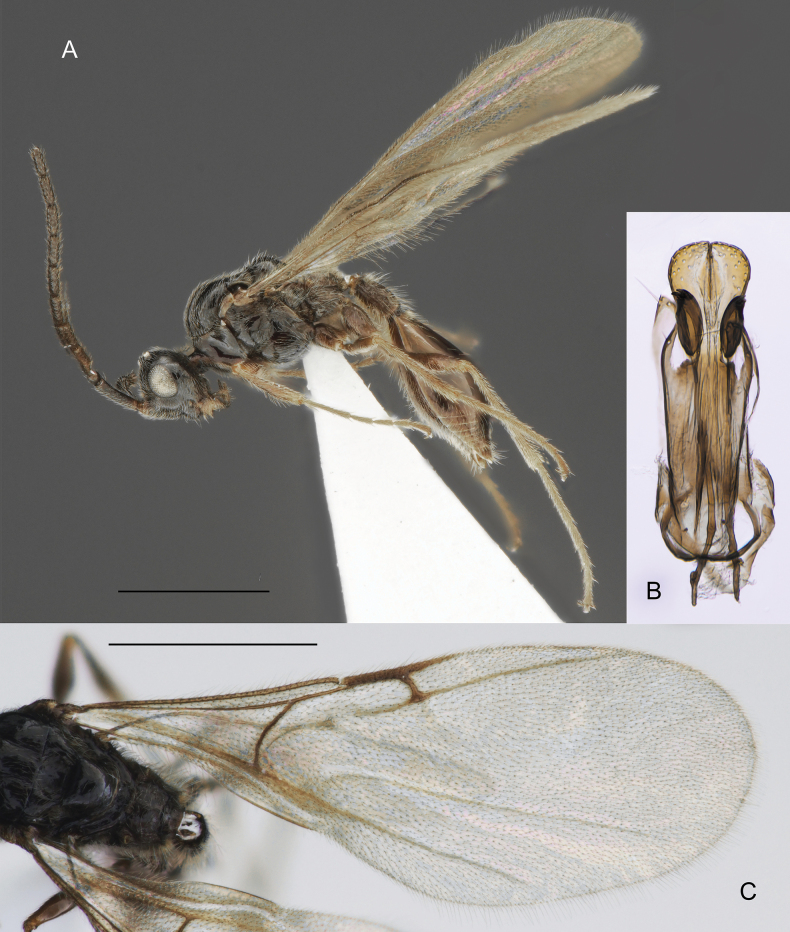
*Zygotavigil* Nixon, male **A** whole insect in lateral view **B** male genitalia **C** fore wing venation. Scale bar: 1 mm.

**Figure 10.**
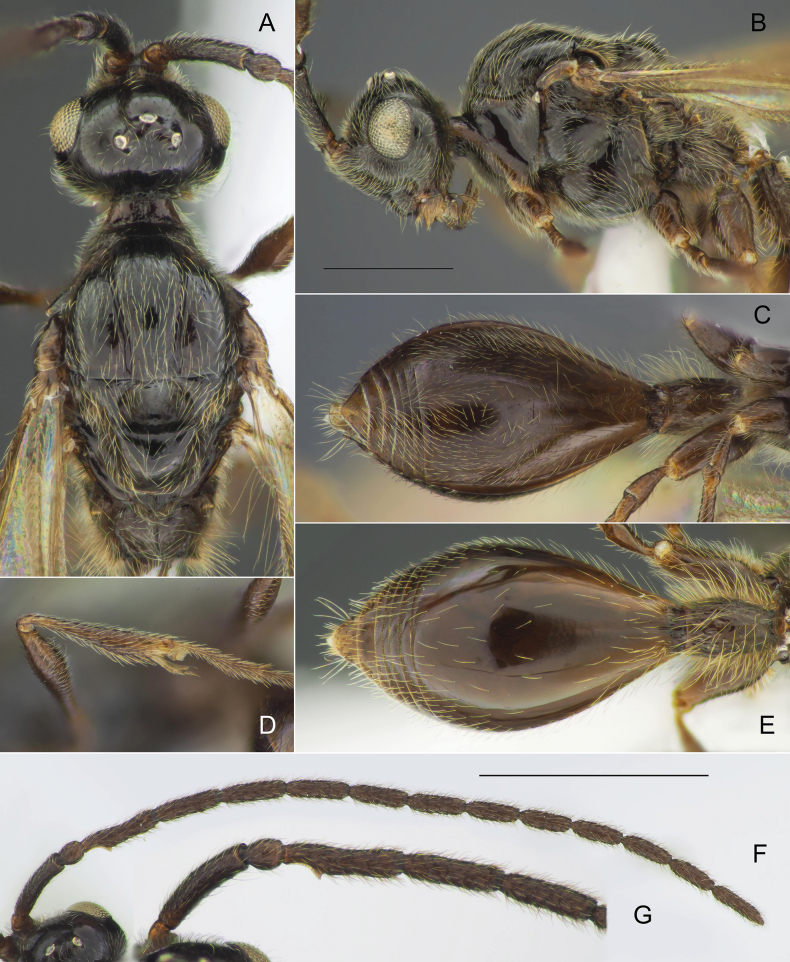
*Zygotavigil* Nixon, details of morphology, male **A, B** head and mesosoma in dorsal (**A**) and lateral (**B**) views **C, E** metasoma, in ventral (**C**) and dorsal (**E**) views **D** fore tibia **F, G** antennae in dorsal view. Scale bars: 0.5 mm (**B**); 1 mm (**F**).

Slender specimens with postmarginal vein clearly shorter than radial cell length (Fig. 9); marginal vein slightly longer than parastigma (Fig. 9C); occipital pit absent; mesopleuron with only small bare area medially (Fig. 10B); axillar depression with scattered setae and only 2 verriculate tubercles; petiole in dorsal view pubescent anteriorly; S2 without micro-puncture sculpture on its anterior half (Fig. 10C); emargination on A3 distinct but not deep, extending to 0.35 of the segment length; fore tibia not modified, entirely pubescent and with several enlarged setae along its inner side (Fig. 10D); petiole with inarticulated elongate carinae (Fig. 10E); base of T2 without lateral corners (Fig. 10E); digitus with two narrow and long spines (Fig. 9B).

#### Distribution.

Europe: Austria, Germany*.

#### Remark.

This species was described by Nixon based on a single male from Austria, but the type of the species was not found (J. Monks pers. com.). Unfortunately, it was not possible to create a BIN from the obtained sequence of the *Zygotavigil* male due to its length (461bp).

### ﻿ Zygota walli sp. nov.

Taxon classificationAnimaliaHymenopteraDiapriidae

E9FDAA78-DF5B-5187-971D-FE20427744D3

https://zoobank.org/DC1B6471-36AC-4653-9044-4D277DFF9DF3

**Figure 11.**
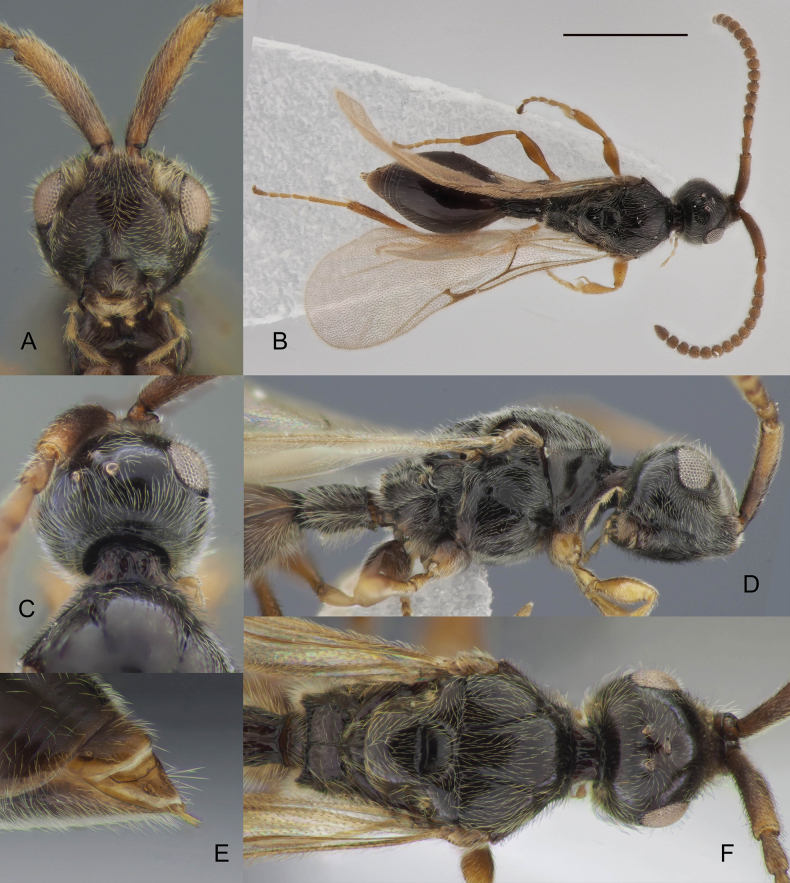
*Zygotawalli* sp. nov. female holotype (ZSMHYM42437-A07) **A** face **B** whole body in dorsal view **C** head, dorsal view **D** head and mesosoma in lateral view **E** apex of metasoma, dorso-lateral view **F** head and mesosoma in lateral view. Scale bar: 1 mm.

**Figure 12.**
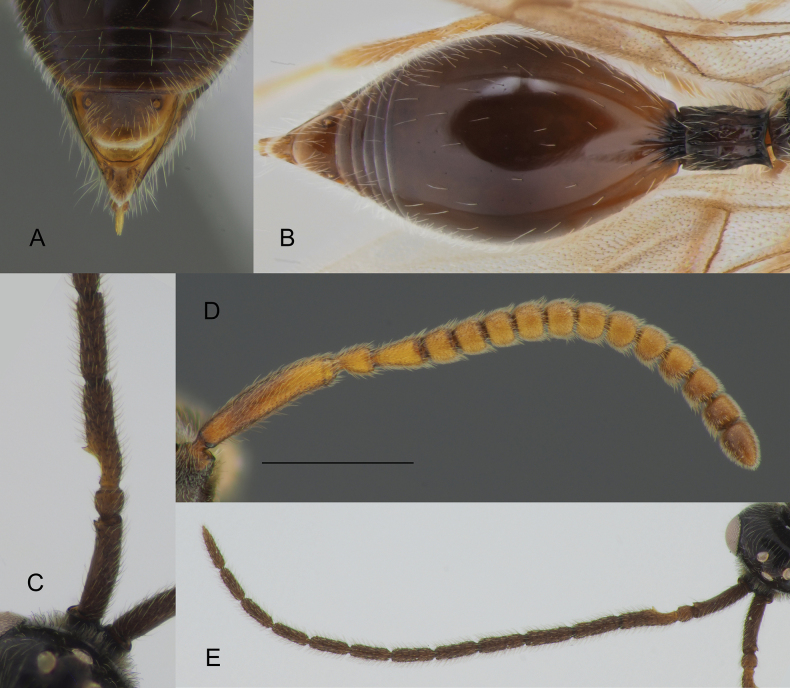
Details of *Zygotawalli* sp. nov. morphology, female (**A, B, D**) and male (**C, D**) **A** apex of metasoma **B** metasoma in dorsal view **C** A1–A4 in dorsal view **D** antenna in lateral view **E** antenna in dorsal view. Scale bar: 0.5 mm.

Figs 1C , 3D, F , 4E , 5A , 11A–F , 12A–E

#### BOLD BIN.

BOLD:ACF9113, BOLD:AER4128.

#### Material examined.

***Holotype*** Germany. BY: Platt, Garmisch-Partenkirchen, 09-Oct-2028, lat. 47.406, long. 11.009, dv.zugsp6.6, ZSMHYM42437-A07, GBOL III leg., BOLD:ACF9113, SNSB-ZSM, 1 ♀.

***Paratypes.*** BY (BOLD:ACF9113): Mittenwald, 13-Jul-2021, 30-Jul-2021, 1 ♀, 2 ♂; Garmisch-Partenkirchen, 05-Jul-2018, 09-Oct-2018, 2 ♀, 1 ♂.

#### Other material.

Germany: BY (BOLD:AER4128): Garmisch-Partenkirchen, 2-Aug-2018 1 ♂; Mittenwald, 30-Jul-2021, 1 ♂; Garmisch-Partenkirchen, 09-Oct-2018, 1 ♂. BY (unsequenced material): Rhoen mountains, 11-Jul-2018, 1 ♂; Oberstdorf, 28-Jun-2016, 1 ♀; Garmisch-Partenkirchen, 13-Aug-2018, 1 ♂.

#### Diagnosis.

**Both sexes**: postmarginal vein distinctly shorter than radial cell length (Figs 3D, 11B); occipital pit absent (Figs 1C, 11C); mesopleuron with only small bare area medially or entirely pubescent (Fig. 11D); axillar depression with scattered setae and only 2 verriculate tubercles; base of T2 with lateral corners (Fig. 12B); S2 with small sculptured area anteriorly (Fig. 4E, green arrow). **Female**: T2 mainly smooth with few scattered micropunctures (Fig. 12B); Т8 with distinct transverse carinae (Fig. 11E, 12A). **Male**: A3 distinctly emarginated (Fig. 12С); fore tibia distinctly modified, broadened with sharp projection and a row of strong setae on the top of it, bare at the apex on its anterior surface (Fig. 3F); digitus armed with 3 teeth (Fig. 5A). *Zygotawalli* sp. nov. differs from all other species mentioned by Macek (1997) in the absence of the occipital pit (Fig. 1C, red arrow).

#### Description.

**Female (*holotype*).** Body length 3.2 mm, antenna length 2 mm, wing length 2.6 mm. Body mainly black with metasoma dark brown; antennae, palpi, mandibles, tegula, legs and venation brown (Fig. 11B).

***Head*** in dorsal view as long (measured with antennal shelf) as wide. Toruli separated from each other by narrow and shallow furrow and from front posteriorly with deep pubescent depression. Ocelli small, OOL twice as long as POL. Eye densely pubescent. Eye diameter 1.2 as long as malar space. Pleurostomal distance as long as malar space. Occipital carina narrow, almost smooth, without occipital pit (Fig. 11C). Head in lateral view as high as long, in frontal view subtriangular, with face smooth and shining. Antennal shelf rugose below toruli in frontal view. Subantennal furrows very short (Fig. 11A). Epistomal sulcus distinct, clypeus convex and smooth. Tentorial pits situated in small hollows. Mandibles not prominent.

***Antennae*** 15-segmented (Figs 11B, 12E). A1 cylindrical, as long as A2–A5 combined, slightly curved, with simple apical rim. A3–A14 as long as wide to slightly transverse: A7–A9 weakly wider than A13–A14. A15 1.7 times as long as wide.

***Mesosoma*** convex, 1.2 times as wide as the head. Pronotal shoulders weakly convex, with transverse carina between them. Epomia with long lower branch and short lateral branch. Lateral part of pronotum strongly impressed, smooth and shining. Mesonotum convex, with percurrent notauli, converging posteriorly. Scutellum convex, smooth, with oval anterior scutellar pit. Axillar depressions smooth, densely pubescence, with a pair of vericulate tubercles. Mesopleuron smooth with deep mesopleural pit, with epicnemial and acetabular bridges (Fig. 11D). Metascutellum with strong median carina and lateral carinas. Metanotal trough smooth and bare. Propodeum slightly transverse, with round posterior rim. Median keel of propodeum simple. Both plicae parallel to each other, slightly projecting posteriorly. Lateral side of propodeum below plicae with lateral longitudinal carina, slightly projecting posteriorly. Fore tibia simple with homogeneous strengthened bristles on the inner side.

***Wings*.** Marginal vein strongly developed, 3.9 times as long as wide (measured medially) and 1.45 times as long as distance from it to basal vein. Radial cell open, radialis long and nebulous (Fig. 11B). Postmarginal vein slightly shorter than stigmal vein; stigmal and postmarginal veins form 65° angle, stigmal vein 0.5 times as long as marginal vein.

***Petiole*** cylindrical, entirely covered with semi-erect pubescence and elongate keels, ventrally with a row of verriculate tubercles. Base of T2 with slightly indicated lateral corners, short medial furrow and straight striation flanked at each side (Fig. 12B). S2 entirely pubescent, base of S2 with group of verriculate tubercles. Apical tergite (T8) with transverse sharp keel (Figs 11E, 12A), smooth and bare anteriorly and smooth and setose posteriorly from the transverse keel.

**Male.** Head distinctly transverse, as wide as mesosoma. Antennae 14-segmented with A4–A14 cylindrical, A3 with keel and emargination extending to 0.35–0.40 of the segment length (Fig. 12C, E). Fore tibia modified, acutely angled on the inner side and covered at the top with several minute bristles (Fig. 3F). Excavation on the fore tibia bare and shining in frontal view. Postmarginal vein 0.5–1.5 times as long as marginal vein (Fig. 3D). Marginal vein 1.3 times as long as distance from it to basal vein or slightly shorter. Petiole 1.5–2.1 times as long as its median width.

#### Etymology.

This newly described species is named after the diapriid taxonomist Ingmar Wall who made himself a name in the Diapriidae research for years.

#### Distribution.

Europe: Germany (Bavaria).

## ﻿Discussion

As a result of our study, new combinations were proposed for 13 of 20 species which have a yet questionable taxonomic position, and two names (*Zygotacaligula* Buhl and *Z.reticulata* Kozlov) were considered synonyms. One species of the genus *Zygota*, *Z.maura* (Kieffer, 1910) remains unstudied and inexplicable. Based on the emarginated fore tibia in males, mentioned in the original description, this species should be without doubt classified in the genus *Zygota* (Kieffer 1910). However, the type specimen of this species has not been found, and the description is not detailed enough to allow further conclusions at the species level or potential synonymies. The types of the two species *Z.strigata* Kozlov, 1978 and *Z.groenlandica* Buhl, 1995 were examined, and both are valid taxa of *Zygota*. *Zygotacilla* Nixon, 1957 and *Z.vigil* Nixon, 1957 were not included in Macek ´s (1997) revision because of the lack of relevant material. Nixon (1957) based both species on a single female (*Z.cilla*) and a single male specimen (*Z.vigil*), yet neither type has been found. The first discovery of a male *Z.vigil* since the description of the species is given here. A female of *Z.cilla*, which is unique in its morphology (Nixon 1957), was not found during this research. Thus, the taxonomic position of all Palearctic species (Johnson 1992, Buhl 1995, 1997, Macek 1997) listed in *Zygota* but not mentioned in Macek ´s (1997) revision, are discussed in this article.

Molecular-based analysis, which was conducted in the framework of this and previous works of GBOL III, has recovered rather poor results for the genus *Zygota* (and others of the Belytinae tribes Cinetini and Belytini; ~68% sequencing success rate) when compared to other diapriid taxa (~90%). Therefore, we recommend future studies invest their efforts into the development of a specific primer set to improve sequencing success. Nevertheless, we significantly improved the amount of genetic information that is available online. Prior to this study, BOLD listed a total of 391 public records that were assigned to 26 BINs globally. Our dataset DS-ZYGPAN presents 178 *Zygota* records and 19 BINs from Germany alone (see also Suppl. material 3).

In this study, some *Zygota* morphospecies were assigned to more than one BIN. This can happen for a variety of reasons: incomplete lineage sorting, heteroplasmy, NUMTs, hybridisation, recent speciation, cryptic species, phylogeographic effects, introgression or endosymbionts or their combinations can influence the outcome of genetically sorting of different OTUs (Raupach et al. 2016). Another factor that plays a key role in the construction of a BIN is the DNA barcoding gap difference between the highest intra- and smallest interspecific variation of a certain taxon. A typical threshold in the genetic distance between two species ranges from 10–15%, but this can vary immensely (Meier et al. 2006, Hebert et al. 2016, Raupach et al. 2016). In our case, 10–15% was indeed a fitting value to delimit species with CO1. A MEGA mean group distance analysis (Suppl. material 3) confirmed our morphological findings, namely, that specimens assigned to the same morphological species all displayed smaller genetic distances between one another than between other morpho-species: *Z.comitans* (mean group distance within all sequences of the BIN: 7%), *Z.spinosa* (5.4%), *Z.parallela* (5.8%), *Z.spinosipes* (6.3%), *Z.ruficornis* (three BINs; 5.3%, 3.5%, 4.3%) and *Z.walli* sp. nov. (2.6%). The corresponding specimens of each BIN cluster together in the taxonomic ML-tree (see Suppl. material 1). An ASAP analysis of the genetic material confirmed the BIN clusters for the genus *Zygota*. The highly variable genus *Pantoclis*, on the other hand, displayed less resemblance when comparing the BINs with ASAP clusters. All of those questionable records were only represented by one or two sequences in our dataset which might explain their uncertain placement.

**Figure 13.**
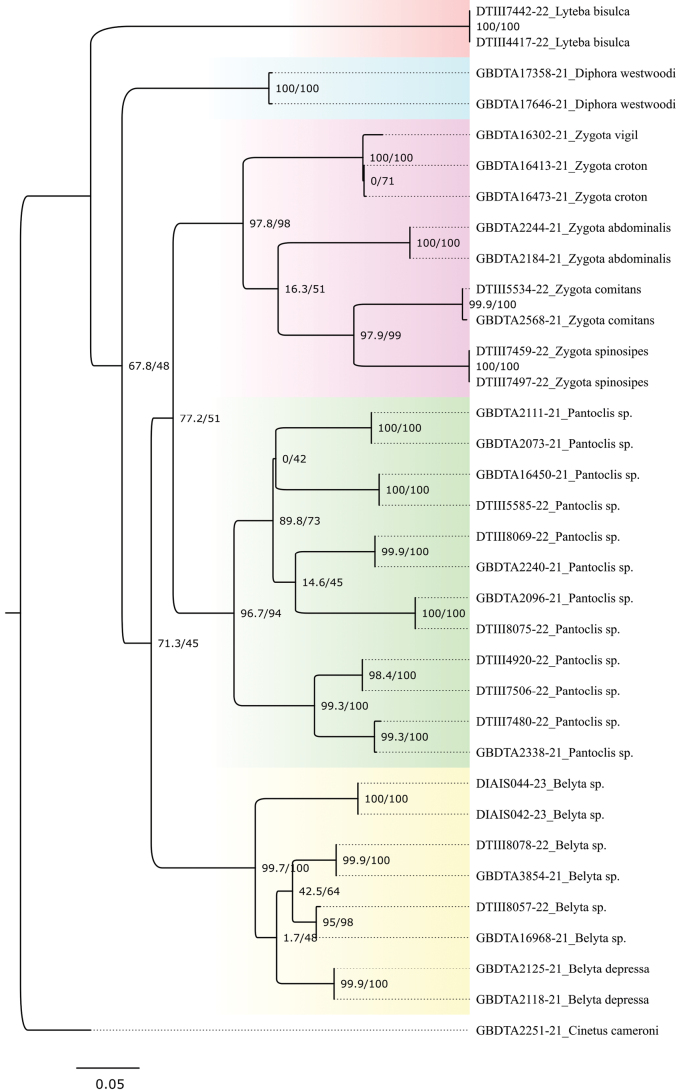
Phylogenetic ML consensus tree of barcoded Belytini specimens with bootstrap/jackknife values and *Cinetuscameroni* as an outgroup.

**Figure 14.**
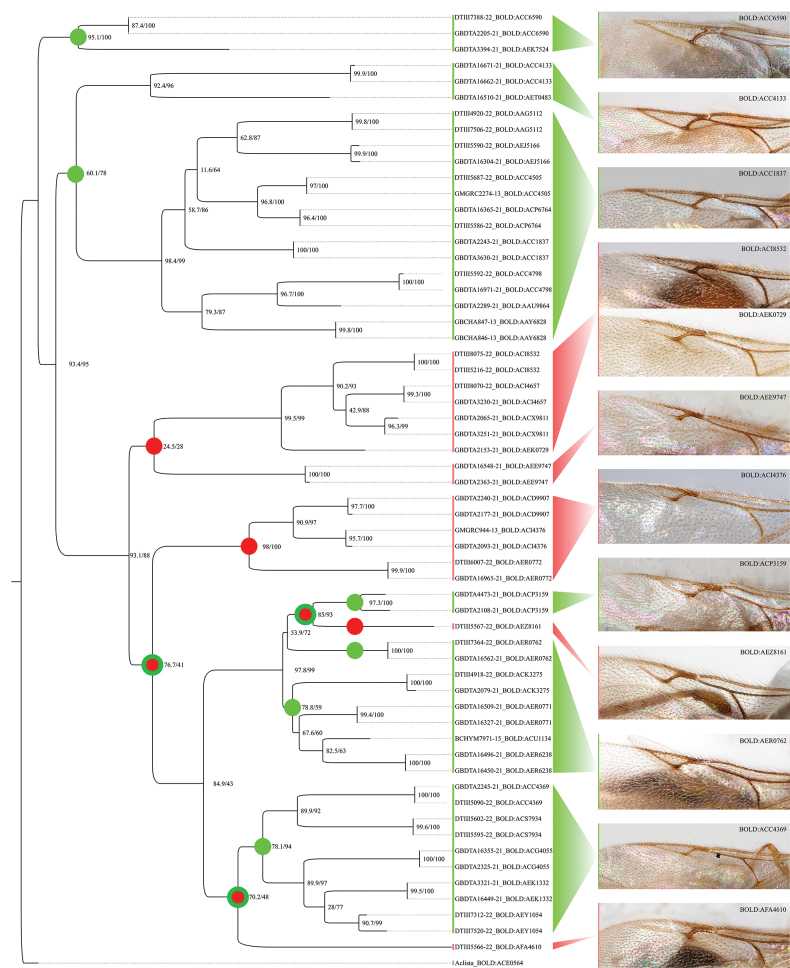
Phylogenetic ML tree of barcoded *Pantoclis* material and the polyphyletic appearance of their wing venation. Green represents the taxa with a closed radial cell while species with an open cell are color-coded red. Each node’s support is displayed by the bootstrap and the jackknife values. *Aclista* was used as an outgroup.

A subset of the available CO1 sequence data of species of the tribe Belytini was used to construct a phylogenetic ML-tree (Fig. 13). Here, the genera *Zygota* and *Pantoclis* were displayed as well-supported sister groups within the Belytini. Fig. 14 shows a more detailed tree with records from all *Pantoclis* BINs we investigated. The data show that some species with an open radial cell are grouped and demonstrate close genetic relationships with species that clearly belong to *Pantoclis* and have a closed radial cell. These findings suggest that the character state of the radial cell reduction cannot be used as an appropriate feature for genus designation. Nixon (1957) also noticed these differences between *Zygota* species and the group of *Pantoclis* species with an open radial cell. He proposed to aggregate them into the *Z.fuscata* – species group “... because of the form of the radial cell and better development of the radialis, this group is transitional between *Pantoclis* and *Zygota* and has perhaps more relationships to the former genus [*Pantoclis*] than to *Zygota* s. str.” (Nixon 1957). Nixon placed six species (*Z.fuscata*, *Z.microtoma*, *Z.striata*, *Z.brevinervis*, *Z.soluta*, *Z.fossulata*) in the *Z.fuscata* – species group which have been transferred to *Pantoclis* here.

In addition, the species transferred to the genus *Pantoclis* in this research are not similar to *Zygota* species in other key characteristics. Unlike *Zygota* species, males of *Pantoclis* never display a modified fore tibia and most of them have slender genitalia with lanceolate apex of aedeagus and a diminished digitus. On the contrary, some *Zygota* males have the digitus with a single strong curved spine, while similar structures are not known for the *Pantoclis* species. All females of *Zygota* show a very short ovipositor, while many *Pantoclis* females (with closed or open radial cell) show a long ovipositor (Fig. 1A). Thus, combining this morphological information with our understanding of the genus *Pantoclis* (see the diagnosis of the genus proposed above), and taking data on the venation variability based on the molecular data into consideration, we propose in this study, new combinations for 13 species previously listed in the genus *Zygota* (Suppl. material 2).

Because a detailed revision of *Pantoclis* is still lacking, it is important to note that the diagnosis presented here is preliminary. The high amount of variation in the morphology and the large species richness of the genus suggest that *Pantoclis* is paraphyletic. On the other hand, as a consequence of the taxonomic changes proposed here, the monophyly of the *Zygota* is now less controversial based on species morphology.

## Supplementary Material

XML Treatment for Pantoclis

XML Treatment for Zygota

XML Treatment for Zygota abdominalis

XML Treatment for Zygota angularis

XML Treatment for Zygota balteata

XML Treatment for Zygota breviuscula

XML Treatment for Zygota claviscapa

XML Treatment for Zygota comitans

XML Treatment for Zygota congener

XML Treatment for Zygota croton

XML Treatment for Zygota excisor

XML Treatment for Zygota nigra

XML Treatment for Zygota parallela

XML Treatment for Zygota praetor

XML Treatment for Zygota pubescens

XML Treatment for Zygota ruficornis

XML Treatment for Zygota sordida

XML Treatment for Zygota spinosa

XML Treatment for Zygota spinosipes

XML Treatment for Zygota vigil

XML Treatment for Zygota walli

## ﻿Additional information

### Author ORCIDs

Jeremy Hübner https://orcid.org/0009-0007-5624-8573

Vasilisa Chemyreva https://orcid.org/0000-0002-6547-6259

Jan Macek https://orcid.org/0000-0003-3104-325X

Viktor Kolyada https://orcid.org/0000-0001-8628-1365

### Data availability

All of the data that support the findings of this study are available in the main text or Supplementary Information.

## Supplementary materials

Supplementary material 1 ML-tree with a subset of all *Pantoclis* and *Zygota* BINs available from our data with one *Aclista* sequence as outgroup Data type png https://binary.pensoft.net/file/1099745This dataset is made available under the Open Database License (http://opendatacommons.org/licenses/odbl/1.0/). The Open Database License (ODbL) is a license agreement intended to allow users to freely share, modify, and use this Dataset while maintaining this same freedom for others, provided that the original source and author(s) are credited.Jeremy Hübner, Vasilisa Chemyreva, Jan Macek, Victor Kolyada

Supplementary material 2 Type information for the taxonomically treated and transferred *Zygota* species Data type xlsx https://binary.pensoft.net/file/1099746This dataset is made available under the Open Database License (http://opendatacommons.org/licenses/odbl/1.0/). The Open Database License (ODbL) is a license agreement intended to allow users to freely share, modify, and use this Dataset while maintaining this same freedom for others, provided that the original source and author(s) are credited.Jeremy Hübner, Vasilisa Chemyreva, Jan Macek, Victor Kolyada

Supplementary material 3 Cluster analyses of the genetic results Data type xlsx https://binary.pensoft.net/file/1099747This dataset is made available under the Open Database License (http://opendatacommons.org/licenses/odbl/1.0/). The Open Database License (ODbL) is a license agreement intended to allow users to freely share, modify, and use this Dataset while maintaining this same freedom for others, provided that the original source and author(s) are credited.Jeremy Hübner, Vasilisa Chemyreva, Jan Macek, Victor Kolyada
