# Supplementary figures and images for: ﻿A review of the genus Zygota (Hymenoptera, Diapriidae) in Germany with taxonomic notes on this genus and its distinction from Pantoclis
Source: Zookeys. 2024 Jul 24;1207:325–53. doi: 10.3897/zookeys.1207.121725 (PMC11292123; doi:10.3897/zookeys.1207.121725)

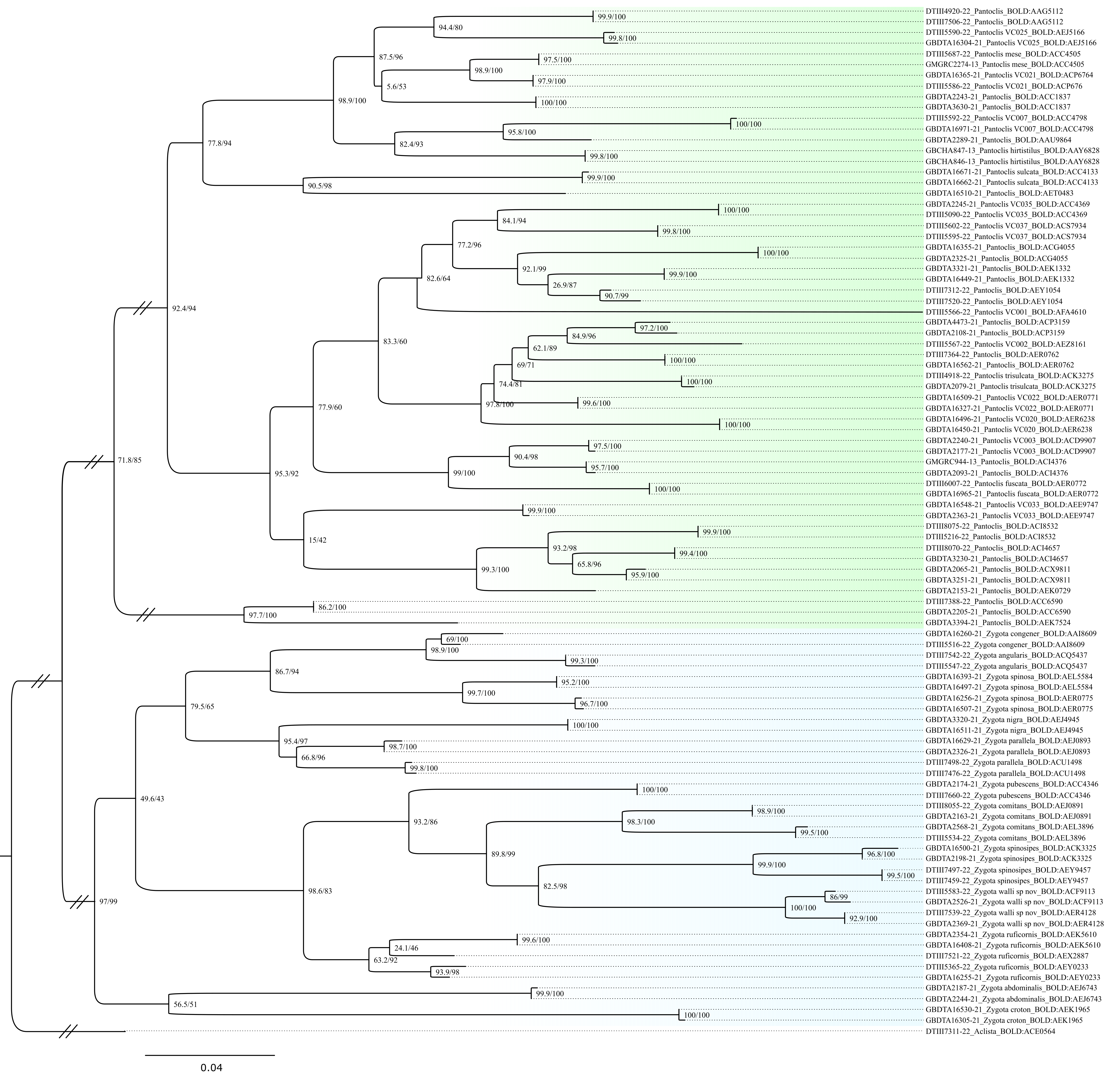

Supplement: Supplementary material 1 — ML-tree with a subset of all Pantoclis and Zygota BINs available from our data with one Aclista sequence as outgroup [file zookeys-1207-325_article-121725__-s001.png]
